# The performance of a new local false discovery rate method on tests of association between coronary artery disease (CAD) and genome-wide genetic variants

**DOI:** 10.1371/journal.pone.0185174

**Published:** 2017-09-20

**Authors:** Shuyan Mei, Ali Karimnezhad, Marie Forest, David R. Bickel, Celia M. T. Greenwood

**Affiliations:** 1 Department of Epidemiology, Biostatistics and Occupational Health, McGill University, Montreal, Québec, Canada; 2 Ottawa Hospital Research Institute, Ottawa, Ontario, Canada; 3 Ottawa Institute of Systems Biology, Faculty of Medicine, University of Ottawa, Ottawa, Ontario, Canada; 4 Department of Biochemistry, Microbiology and Immunology, University of Ottawa, Ottawa, Ontario, Canada; 5 Lady Davis Institute for Medical Research, Jewish General Hospital, Montreal, Québec, Canada; 6 Department of Mathematics and Statistics, University of Ottawa, Ottawa, Ontario, Canada; 7 Department of Oncology, McGill University, Montreal, Québec, Canada; 8 Department of Human Genetics, McGill University, Montreal, Québec, Canada; University of the Chinese Academy of Sciences, CHINA

## Abstract

The maximum entropy (ME) method is a recently-developed approach for estimating local false discovery rates (LFDR) that incorporates external information allowing assignment of a subset of tests to a category with a different prior probability of following the null hypothesis. Using this ME method, we have reanalyzed the findings from a recent large genome-wide association study of coronary artery disease (CAD), incorporating biologic annotations. Our revised LFDR estimates show many large reductions in LFDR, particularly among the genetic variants belonging to annotation categories that were known to be of particular interest for CAD. However, among SNPs with rare minor allele frequencies, the reductions in LFDR were modest in size.

## Introduction

Current technologies for measuring genome-wide genetic variation easily capture millions of variants across the genome. High dimensional genotyping arrays already commonly include several million variants. Through direct sequencing or by imputation against large previously-sequenced reference panels where sequencing has been performed[1–3], the number of assayed genetic variants may increase substantially. Hence, when performing an association study to identify genetic variation associated with a phenotype of interest, the number of variants to be tested may easily include many millions of variants[4], most of which will be single nucleotide polymorphisms (SNPs).

Stringent genome-wide significance thresholds of 5x10^-8^, established to control the family-wise error rate (FWER) at approximately 5% for genome-wide testing, have been in standard usage in the field of human genetics for many years[5]. Recently, this threshold has been refined downwards to account for the much larger number of variants tested in sequenced or imputed data[6]. Certainly, strict application of these thresholds has led to substantially increased reproducibility of identified genetic associations[7–9].

However, although few positive results are now published when associations meet genome-wide significance thresholds controlling FWER, power can be severely compromised by the use of these necessarily very small significance thresholds. Substantial missing heritability has been seen for many traits and diseases, yet many true loci of small effect may not be identified due to low power studies.

In order to improve power, many research groups are increasing their sample sizes through collaborations and meta-analyses (e.g.[3]). Other groups are pursuing analytic alternatives that relax the genome-wide significance threshold. In particular, strategies that control the false discovery rate (FDR) instead of the family-wise error rate (FWER) often allow testing at much more liberal significance thresholds[10, 11]. The argument can be made that when performing millions of tests, that to control the probability of at least one false positive result at 5% is unnecessarily strict, and that it makes sense to control merely the proportion of false results among the set of significant results, i.e. the FDR.

**Fig 1:**
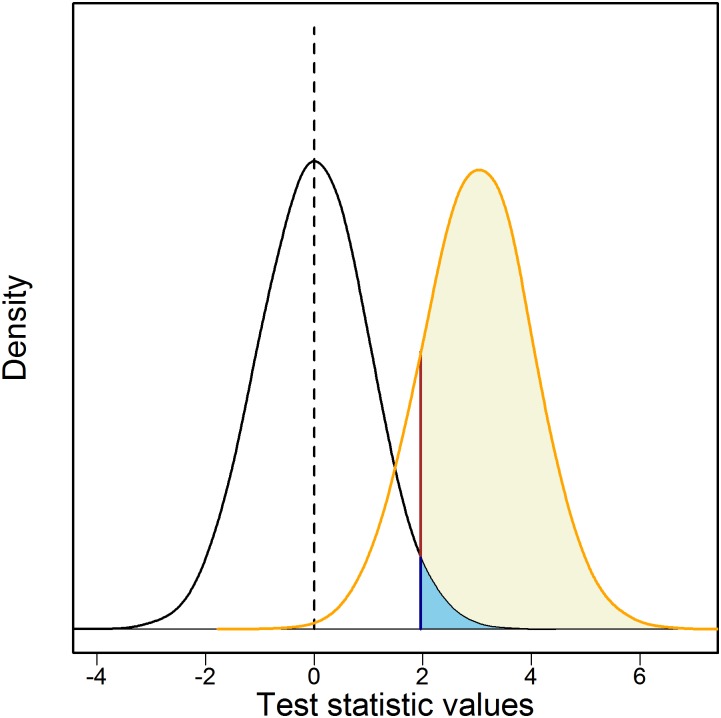
FDR and LFDR. FDR at a cutoff of 1.95 (p-value 0.05 for a normally distributed test) is the ratio of the area of the light blue region divided by the area of (beige plus light blue). LFDR compares the height of the dark blue line to the height of the brown line.

Despite the potential benefits of using FDR-defined significance thresholds instead of the FWER-defined thresholds, control of type 1 errors through the use of a chosen, fixed FDR threshold may be suboptimal. In fact, the use fixed FDR thresholds incurs a bias and tends to allow a higher proportion of false positives than indicated by the selected FDR[12]. Therefore, a better strategy may be to rely on the local FDR (LFDR), which is the probability of a test result being a false positive, given the exact value of the test statistic[10] (Fig 1).

Power remains low even when FDR methods (or LFDR methods) are used, due not only to the chosen significance thresholds, but also because the effect sizes of most genetic variants tend to be small. Furthermore, power is particularly low for variants that have lower minor allele frequencies—i.e. variants that are uncommon in the population—since the standard errors associated with the estimated effects are large due to the small number of individuals carrying the uncommon variants. Additional strategies for increasing power are, therefore, of great interest. Approaches motivated along these lines include finding subsets of genetic variants that are of particular interest, and performing significance threshold adjustments that prioritize these subsets. It has been shown that judicious use of good external annotation about the genetic variants can increase the statistical power to identify associations with low prior power, and that the significance rankings can be improved[13, 14]. For example, recent studies have shown that some functional categories of the genome contribute disproportionately to the heritability of complex diseases.[15, 16] An approach has been developed, using mixed linear models, to systematically leverage annotation information together with genome-wide genotype data to identify subset of SNPs that show significant heritability-enrichment[17, 18].

In this paper, we focus on obtaining improved false discovery rate estimates for coronary artery disease (CAD) through the use of an LFDR method that incorporates external information about the genetic variants, leading to a posterior probability of non-association that varies with the annotations. Similar approaches for the FDR, i.e. methods that stratify or modify the FDR as a function of external information, have been shown to be effective in reducing the overall type 1 error rates[13, 19, 20]. Specifically, we implement a new LFDR estimation method recently developed by David Bickel’s group at University of Ottawa: the ME method[21], that optimally combines LFDR estimates from a small class of test statistics (for some genetic variants) with the larger set of all tests[21]. The theoretical framework underlying the ME LFDR method has been provided in the Appendix. Here, the small class is defined by external annotation categories that showed significant enrichment of CAD heritability.

We modelled the p-values arising from the CARDIOGRAMplusC4D genome-wide association (GWA) consortium[3] to explore the performance of these new false discovery rate estimators. We selected nine functional categories where heritability of CAD is known to be enhanced to define high risk subsets of SNPs[22] and we compare LFDR results with and without the use of external annotation information to demonstrate the potential benefits.

## Methods

### CARDIOGRAM

The p-values from the CARDIOGRAMplusC4D Consortium (http://www.cardiogramplusc4d.org/data-downloads/) meta-analysis GWA study of coronary artery disease[3] (CAD) were extracted for our investigations here. CARDIOGRAMplusC4D included 60,801 cases and 123,504 controls from 48 studies, and tested for association at 9,455,779 variants. We summarize briefly here the methods used to calculate these p-values; detail can be found in the primary publication of CARDIOGRAMplusC4D[3]. Imputation was based on the 1000 Genomes phase 1 version 3 training set with 38 million variants of which over half are low frequency (MAF < 0.005) and one-fifth are common (MAF > 0.05) variants. After selecting variants that surpassed allele frequency (MAF > 0.005) and imputation quality control criteria in at least 29 (>60%) of the studies, 8.6 million SNPs and 836K (9%) indels were included in the meta-analysis; of these, 2.7 million (29%) were low frequency variants (0.005 < MAF < 0.05). The tests of significance arising from the meta-analysis, after application of genomic control, were used for our investigations of LFDR.

### LFDR estimation with maximum entropy method

There are several well-known approaches to estimate LFDR[23–28]. In this paper, we used the recently-developed ME estimation method[21]. In this method, we assume we have several categories of SNPs where the categorization is obtained from external annotation. Each SNP may be a member in more than one category or reference set. The ME procedure first calculates the LFDR in each of the reference sets. For the SNPs in the intersection of two reference sets, there will therefore be two estimates of LFDR, and the concept of maximum entropy is then used to obtain a single estimate.

To decide whether a separate or a combined reference class should be used, the ME method bases the LFDR estimate mostly on the separate reference class if it has enough SNPs for reliable estimation and otherwise uses the combined reference class alone [21, 29]. ME is so-called because it minimizes the relative entropy function over a confidence interval or likelihood interval constructed based on the separate reference class. If the interval is sufficiently narrow, the separate reference class has enough SNPs to derive reliable estimates of LFDR. In this case, the ME method chooses the separate reference class to estimate the LFDR, and the LFDR estimate is the limit of the interval that is closest to the estimate based on the combined reference class. On the other hand, if the constructed interval is so wide that it includes the estimate of the LFDR based on the combined reference class, then the separate-class estimate is considered unreliable. In that case, the ME method estimates the LFDR based solely on the combined reference class.

### Construction of reference sets

SNPs were categorized into 53 overlapping functional categories based on the annotation data from Finucane et al. [15] and their polygenic contributions to heritability of CAD were estimated using mixed linear models[15, 17, 22]. Nine functional categories showed significant heritability enrichment (Bonferroni corrected P<0.01). These categories were used to illustrate the performance of the LFDR method. For ease of presentation, results for three categories (Hoffman enhancers, H3 lysine 9 acetylation (H3K9ac), and fetal DNAse I hypersensitivity (DHS) mark (H3K27ac)) are highlighted in the main paper and additional results are in the Supplement.

## Results

The results from CARDIOGRAMplusC4D can be seen in their primary publication[3]. Here, we used the p-values obtained at 9.45 million variants to estimate LFDR. We define three different levels of “significance” for use in our explorations. Firstly, there are 1,836 SNPs that met the most stringent threshold of p-values less than 1x10^-8^ (Table 1)[6], appropriate for genome-wide sequencing studies and MAFs down to 0.005. There are 2,213 SNPs with p-values less than 5x10^-8^, and 32,508 that are in the tail of the QQ plot that deviates from the null distribution. For this latter definition, we refer to these SNPs as “P deviated” in Table 1, and the significance threshold is 0.001 (i.e 3 on the – log 10 scale). The gene names (if available), p-values, parameter estimates and MAF for each of the p-deviated SNPs are provided in S1 Table.

**Table 1:** Number of SNPs by minor allele frequency bins, as well as the number and percentage of significant SNPs, using several definitions of statistical significance.

MAF bins	<0.001	0.001–0.005	0.005–0.01	0.01–0.05	≥0.05	All
**# SNPs**	0	0	240,423	2,500,103	6,715,230	9,455,778
**(%) of row**	0	0	2.54	26.44	71.02	100
P deviated*	0	0	103	1,988	30,417	32,508
**(%) of row**	0	0	0.32	6.11	93.57	100
**P<5x10**^**-8**^	0	0	0	61	2,152	2,213
**(%) of row**	0	0	0	2.76	97.24	100
**P<1x10**^**-8**^	0	0	0	39	1,836	1,875
**(%) of row**	0	0	0	2.08	97.92	100

*P deviated: the p-value was in the tail of the QQ plot, after a point of inflexion where the line sloped away from the line of expectation. This includes all SNPs with p< 0.0074

Table 1 also shows the proportion of the significant SNPs—by each definition of significance—that fall into different MAF bins. SNPs with MAF≥0.05 account for 71% of all analyzed SNPs, but they account for 93.6% of the p-deviated set. To examine these data from another perspective, SNPs with a frequency less than 1% account for 2.5% of analyzed SNPs but only 0.32% of deviated SNPs. Therefore, the data indicate that there may be too few SNPs showing statistical evidence of association at small MAFs, probably due to low power.

**Fig 2:**
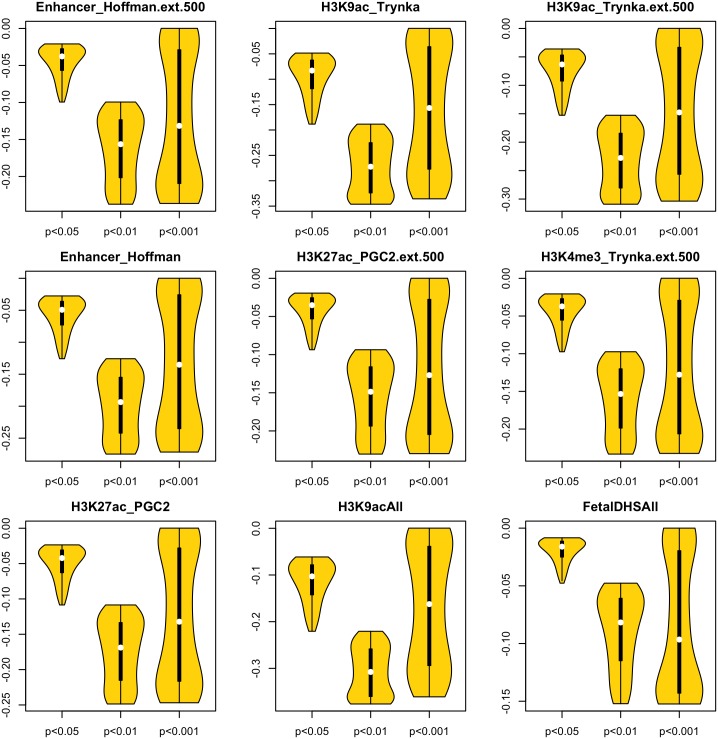
Changes in LFDR estimates between unadjusted LFDR and LFDR estimated with the ME method, when each of nine functional annotations are used to define a high risk subset of SNPs. Within each panel, the three distributions are divided by p-value ranges: unadjusted p<0.05; unadjusted p<0.01; unadjusted p<0.001.

**Fig 3:**
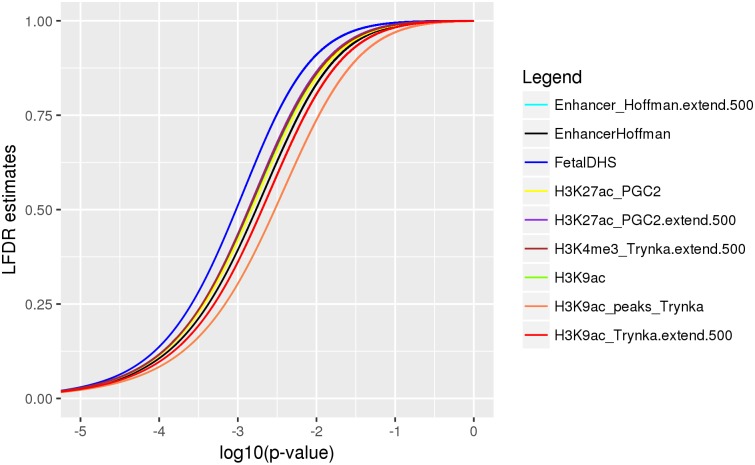
LFDR estimates with the ME method, as a function of the –log(10) of the raw p-values, for all nine SNP annotation categories considered.

To demonstrate the potential of the ME LFDR method, we applied it to nine annotation categories(22) known to significantly contribute to CAD heritability (Table 2). Changes of LFDR estimates for all nine categories are shown with violin plots in Fig 2. For any of the annotations, the largest decreases in the LFDR estimates can be found among the set of p-values less than 0.01, where a majority of the SNPs show substantial decreases in their LFDR estimates using the ME method, with any of the three annotation categories. For smaller p-values (<0.001), the magnitudes of the changes in LFDR estimates are spread quite uniformly across the possible range. For the Fetal DHS annotation (bottom right in Fig 2), which was the least significant LD-score enriched category in Table 2, LFDR decreased by at least 10% at only 0.66% 4785/722,377 of the SNPs when we used the ME method. However, SNPs that showed substantial decreases in LFDR were more common for some of the other annotations. For example, we found a 20% decrease in LFDR for 0.85% of Hoffman enhancer SNPs (4,530 / 533,446), and a 30% decrease in LFDR for 0.99% of H3K9ac histone modifications (3200 /322,804). Fig 3 displays the LFDR estimates versus the p-values after using the ME method, and demonstrates that the benefit associated with the ME method is strongest for the H3K9ac category, whether with the extended window, or with post-processing following [30]. In agreement with Fig 2, it can be seen that the largest LFDR estimates are found for the Fetal DHS annotation.

**Table 2:** Observed heritability (h^2^ obs) and its standard error (SE), expected heritability (h^2^ exp) and the adjusted P-value from LD-score regression for enrichment in CAD. Also, the distances between p-value distributions (D-statistics) from Kolmogorov-Smirnov tests are shown, comparing different MAF groups: (a) [0.005–0.01) vs. [0.01–0.05); (b) [0.005–0.001) vs. (≥0.05); (c) [0.01–0.05) vs. (≥0.05).

Annotation Category	h^2^ obs (SE)	h^2^ exp	P-value (adjusted)^(1)^	# of SNPs ^(2)^	KS-test D measure (a,b,c)
Enhancer_Hoffman. extend.500^(3)^	0.18 (0.03)	0.03	1.1x10^-04^	401,897	0.030, 0.069, 0.042
H3K9ac_Trynka	0.15 (0.03)	0.02	2.7x10^-04^	322,412	0.027, 0.074, 0.048
H3K9ac_Trynka.extend.500	0.18 (0.03)	0.04	3.7x10^-04^	601,848	0.028, 0.072, 0.045
Enhancer_Hoffman	0.14 (0.03)	0.01	4.1x10^-04^	163,480	0.030, 0.072, 0.044
H3K27ac_PGC2.extend.500	0.19 (0.03)	0.07	3.8x10^-03^	962,593	0.024, 0.065, 0.041
H3K4me3_Trynka.extend.500	0.20 (0.04)	0.05	3.9x10^-03^	713,844	0.024, 0.065, 0.042
H3K27ac_PGC2	0.18 (0.03)	0.05	3.9x10^-03^	768,410	0.024, 0.065, 0.042
H3K9ac_peaks_Trynka	0.11 (0.03)	0.01	4.0x10^-03^	95,531	0.032, 0.079, 0.049
FetalDHS_Trynka	0.18 (0.04)	0.02	9.1x10^-03^	255,582	0.022, 0.059, 0.039

^(1)^ Adjusted p-value for enrichment, using a Bonferroni correction

^(2)^ The number of SNPs used for the adjusted p-value

^(3)^ “extend.500” implies that a 500 base pair window around the category was included with the annotation to minimize inflation of heritability from flanking regions[22]

**Fig 4:**
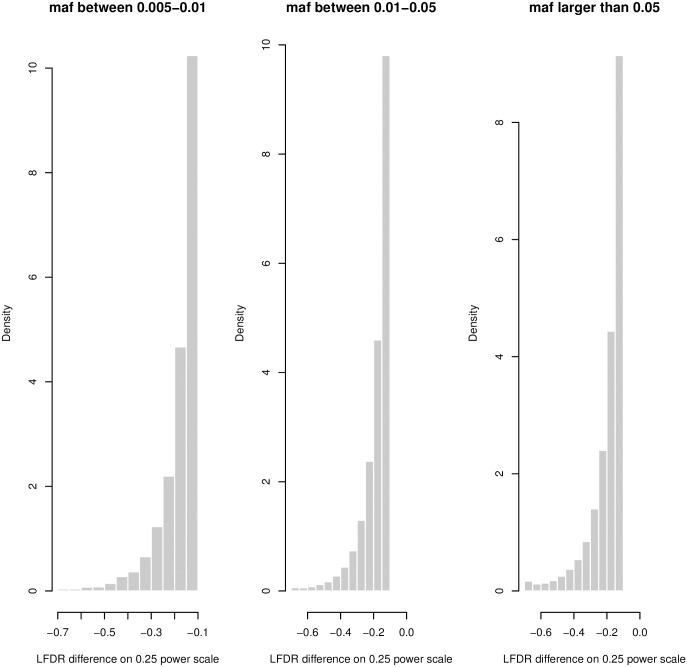
Histogram of LFDR differences for three MAF categories using the Enhancer Hoffman.extended.500 annotation. Differences are on an 0.25 power scale. (Left) MAF between 0.005 and 0.01. (Middle) MAF between 0.01 and 0.05. (Right) MAF greater than 0.05.

In Fig 4, the changes in LFDR for the Enhancer Hoffman (extended 500bp) annotation are shown as a function of MAF. Although the differences are not very discernible to the eye, the distribution of changes in LFDR is more left-skewed when the MAFs are smaller. Kolmogorov-Smirnov (KS) tests were used to compare the LFDR distributions between MAF groups for all nine functional categories (Table 2). All tests were highly statistically significant (p<10^−16^) indicating differences between the distributions. Table 2 shows that the magnitudes of the distances between distributions in different MAF subgroups, as measured by the D-statistic of the KS tests, are quite consistent across the annotations. The general pattern is that LFDR values tend to be smaller for the SNPs in the groups with smaller MAFs, i.e. the LFDR empirical cumulative distribution (ECDF) for SNPs with MAFs in (0.005–0.01) is shifted to the right (i.e. lower values) than the ECDF for SNPs with MAFs in (0.01–0.05) or SNPs with MAF>0.05. We note that since the KS test is rank based, identical test results are obtained when using p-values or LFDR estimates.

**Fig 5:**
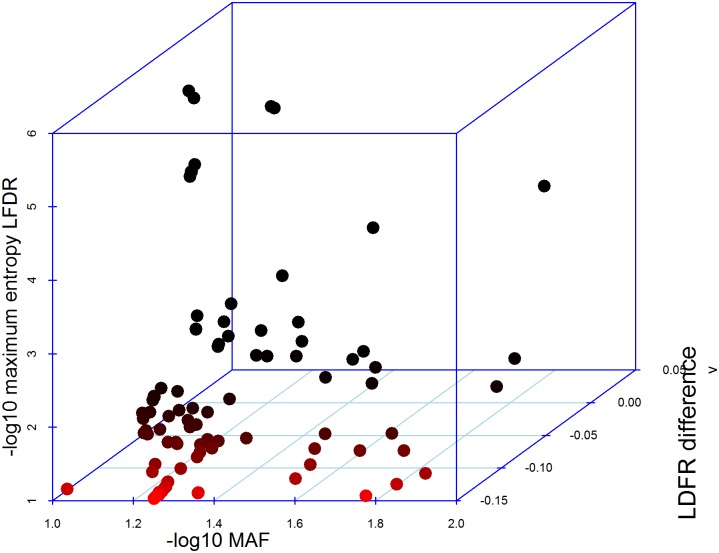
Scatter plot of the LFDR-ME estimates by minor allele frequency and the decrease in LFDR estimates using the ME method, when using the H3K9ac annotation.

Finally, in Fig 5, we focus on SNPs with small MAF (<0.10) and with small LFDR estimates (LFDR-ME<0.10) for H3K9ac, where only 93 SNPs are selected by this filter. For comparison, in S1 and S2 Figs, similar results are shown for Enhancer Hoffman and Fetal DHS, where 67 SNPs were selected by the filter for Enhancer Hoffman, and 63 for Fetal DHS). Three-dimensional scatterplots show the relationships between LFDR, MAF and the LFDR change in these subsets of SNPs. In fact, the SNPs with the larger decreases in their LFDR estimates tend to be those with larger MAF, and furthermore, the SNPs with the smallest local false discovery rates tend to have MAF closer to the upper bound of 0.10 and to show have only small decreases in their LFDR with the new method.

Among the selected subset of 93 SNPs from the H3K9ac annotation, rs41423244 on chromosome 12 showed the greatest LFDR decrease of 14.7%, from 24.7% to 9.998%, with a raw p-value of 1.35x10^-4^. This SNP lies in the CS gene (citrate synthase), and the gene has been previously associated with psoriasis, height and celiac disease. Similarly, the SNP with the largest LFDR decrease among the 67 SNPs highlighted in S1 Fig (Enhancer Hoffman) is rs61877912, which is located on chromosome 11 in an H3K27ac mark in gene *DENND5A*. The naïve p-value is 8.76x10^-5^; LFDR falls from 18.5% to 9.5% with the ME method. Although no previous GWAS associations have been reported with this SNP, the gene DENND5A has been associated with Beta2-glycoprotein plasma levels. Finally, the SNP whose LFDR was most influenced by use of the fetal DHS mark is rs75274818 on chromosome 12 (naïve p = 6.1x10^-5^; LFDR.ME 9.9%; original LFDR 14.5%), located in *SLC39A5*, a zinc transporter. Again no previous GWAS associations have been reported with this SNP, but this gene has been associated with inflammatory skin disease and height.

## Discussion

These data showed many associations with CAD as has been previously reported[3]. However, since power to detect associations with rare genetic variants is usually low, our goal here was to investigate the potential improvements in power associated with a new LFDR method that incorporates external SNP annotation.

Although the LFDR estimates changed for many SNPs, the LFDR estimates that changed most due to the use of the ME method were not those that were particularly rare. When we restricted SNPs to those with MAF <0.1 and LFDR < 0.1, it was always the SNPs near the upper MAF bound that had the largest LFDR decreases (Fig 5). It seems that the ME method is improving the power to detect SNPs with p-values that are small; however, among SNPs that demonstrated genome-wide significance, there were few with small MAFs.

Here, we explored the effect on LFDR estimates by partitioning SNPs into reference sets using functional annotation categories pointing towards excess risk for CAD that were obtained from LD-score regression[15]. The approach that led to this categorization of the SNPs leverages linkage disequilibrium patterns and known regulatory features, and then partitions the heritability for GWAS summary statistics while accounting for linkage disequilibrium patterns. The process or strategy for determining the best reference class of SNPs is an example of what is known as the *reference class problem*; see [31] for references. In general, the potentially-greater relevance of smaller reference classes must be balanced against their greater variability, as Efron[32] discussed (see also Section 10.4 of [33]). The maximum entropy method is an attempt to automatically achieve that balance. The ME method can be applied to many other ways of defining reference classes. For example, a reference set could be derived from prior evidence for association in the region [13, 19], or many different kinds of functional annotation. For coding variants, reference sets could be based on whether amino acids are likely to be affected by a nucleotide change [34, 35]. Annotation can also be based on whether the SNPs in the set are themselves located in regulatory features or demonstrate conservation across species[36]. Here, the annotation definition depends not only on whether the SNPs themselves are annotated, but also whether the SNPs are in linkage disequilibrium with an interesting annotation, which allows enlargement of the featured reference class. However, in more generality, LFDR estimates can depend on many kinds of additional information that allow definition of classes of variants, and the ME method, in particular, is applicable when there is uncertainty about which reference class should be used.

The general concept of giving different priority to different subsets of hypotheses has been previously approached in several ways. Stratified FDR estimates can be obtained by separately calculating the FDR in different classes, and then combining the results[13, 19]. The weighted FDR method assigns an externally-chosen weight to each test[37], and the prioritized subset method[38] identifies a subset of SNPs expected to show stronger significance when calculating FDR. Unlike these methods, ME is designed for estimation of LDFR. Due to the balancing built into the ME method, if the chosen subsets are not optimal, then the LFDR estimate will be obtained from the larger reference class, hence providing protection against a poor choice of annotation.

Some substantial decreases in LFDR were seen in our work—as large as a drop of 0.4 in the LFDR estimate. Inevitably, these very large changes tended to occur for SNPs where the original LFDR estimate was large, and hence these SNPs may not be of great interest. Nevertheless, the ME LFDR method has the potential to increase the level of interest for pursuing a SNP for further investigations by using external annotation in a statistically principled way, and we saw larger reductions in the LFDR. Since all the LFDR estimates calculated here are relevant for functional annotations shown to be significantly associated with CAD, SNPs associated with substantially reduced LFDR estimates may be worth further investigation. Therefore, we have provided a spreadsheet (S1 Table) for all deviated SNPs indicating the LFDR estimates for each of the nine annotation categories.

## Appendix

Following [39] the ME method is derived as follows. The method assumes that the distribution of test statistics, *t*_*i*_, follows a chi-square distribution. Under the null hypothesis that there is no association between SNP *i* and the disease, the test statistic *t*_*i*_ follows the central chi-square distribution with one degree of freedom and the corresponding density is denoted by *g*_0_(.). Under the alternative hypothesis, the test statistic *t*_*i*_ is assumed to follow a non-central chi-square distribution with one degree of freedom and non-centrality parameter *δ*. We refer to the corresponding density by *g*_*δ*_(.). Now, let

$$\psi_{i}=\frac{\pi_{0}g_{0}(t_{i})}{\pi_{0}g_{0}(t_{i})+(1-\pi_{0})g_{\delta }(t_{i})}$$

be the LFDR based on observing test statistic *t*_*i*_, where *π*_0_ is prior probability of SNPs not being associated with the disease. To estimate *ψ*_*i*_, one would have to estimate the parameters *π*_0_ and *δ*. If SNP *i* belongs to both the separate and combined reference classes, the estimation procedure can be based on either a small or a combined reference class selection. For such a SNP, working only with those SNPs that belong to the separate reference class *S*, one can get ${\hat{\pi }}_{S}$ and ${\hat{\delta }}_{S}$, as estimates of parameters *π*_0_ and *δ*. Replacing the estimated values ${\hat{\pi }}_{S}$ and ${\hat{\delta }}_{S}$ in the above equation leads to ${\hat{\psi }}_{i,S}$. Alternatively, assuming that all SNPs belong to a combined reference class *C*, one might get ${\hat{\pi }}_{C}$ and ${\hat{\delta }}_{C}$, as estimates of parameters *π*_0_ and *δ* which results in ${\hat{\psi }}_{i,C}$. Obviously, ${\hat{\psi }}_{i,S}$ might differ from ${\hat{\psi }}_{i,C}$. In this case, one needs to make a careful decision regarding the choice of a reference class.

Following [39], a likelihood set needs to be constructed. To do so, for all SNPs belonging to the separate reference class *S*, define the following likelihood set

$$L_{S}=\left\{\tau :\frac{L(\tau )}{L({\hat{\tau }}_{S})}\geq \frac{1}{2^{a}},\pi_{0}\;\in \;[0,1],\;\delta \;\in [d_{1},\;d_{2}]\right\},$$

where *τ* = (*π*_0_, *δ*), *L*(*τ*) = Π_*i*_(*π*_0_*g*_0_(*t*_*i*_) + (1 − *π*_0_)*g*_*δ*_(*t*_*i*_)) is the likelihood function based on SNPs falling into the separate reference class *S*, *a* is a pre-determined threshold, and *d*_1_ and *d*_2_ are pre-specified limits of the non-centrality parameter *δ*, and ${\hat{\tau }}_{S}=\left({\hat{\pi }}_{S},{\hat{\delta }}_{S}\right)$ is maximum likelihood estimate of *τ*, i.e. ${\hat{\tau }}_{S}=\text{arg }{\text{max}}_{\pi_{0}\;\in \;[0,1],\;\delta \;\in [d_{1},\;d_{2}]}L(\tau )$. According to [39], we chose *a* = 3, *d*_1_ = 0.1 and *d*_2_ = 50.

The likelihood set *L*_*s*_ provides a set of pairs of (*π*_0_, *δ*) that satisfy the condition $\frac{L(\tau )}{L({\hat{\tau }}_{S})}\geq \frac{1}{2^{a}}$. For such a pair of (*π*_0_, *δ*), a value of *ψ*_*i*_ can be computed. Computing *ψ*_*i*_ values for all pairs of (*π*_0_, *δ*) of *L*_*s*_ would provide us a range of LFDR values, say $\left[\psi_{i}^{L},\psi_{i}^{U}\right]$. Now, for each *ψ*_*i*_, consider the following relative entropy function

$$D(\psi_{i},{\hat{\psi }}_{i,C})=\psi_{i}\text{ log}\left(\frac{\psi_{i}}{{\hat{\psi }}_{i,C}}\right)+(1-\psi_{i})\text{ log}\left(\frac{1-\psi_{i}}{1-{\hat{\psi }}_{i,C}}\right).$$

Then *ψ*_*i*,*ME*_, the ME estimate, is the value of *ψ*_*i*_ that minimizes the relative entropy function $D(\psi_{i},{\hat{\psi }}_{i,C})$ over the interval $\left[\psi_{i}^{L},\psi_{i}^{U}\right]$. By the above procedure, if ${\hat{\psi }}_{i,C}\in \left[\psi_{i}^{L},\psi_{i}^{U}\right]$, then $\psi_{i}{{}_{,}}_{ME}={\hat{\psi }}_{i,C}$. Otherwise, if ${\hat{\psi }}_{i,C}<\psi_{i}^{L}$, then $\psi_{i}{{}_{,}}_{ME}=\psi_{i}^{L}$ and if ${\hat{\psi }}_{i,C}>\psi_{i}^{U}$, then $\psi_{i}{{}_{,}}_{ME}=\psi_{i}^{U}$. For technical details, readers may refer to [39].

## Supporting information

S1 Fig Scatter plot of the LFDR-ME estimates by minor allele frequency and the decrease in LFDR estimates using the ME method, when using the Enhancer Hoffman annotation. (TIF)

S2 Fig Scatter plot of the LFDR-ME estimates by minor allele frequency and the decrease in LFDR estimates using the ME method, when using the Fetal DHS annotation. (TIF)

S1 Table Local false discovery rate estimates using the maximum entropy method for nine annotation categories. Columns include the SNP id (legendrs), chromosome (chr), position (pos), minor allele frequency (maf), slope coefficient (beta) and p-value (p_dgc) for association with CAD from the consortium, z-squared (z_sq), and then various LFDR estimates. They are named for the set of SNPs used (LFDR.ME for the ME method, LFDR.Big for LFDR estimated from the large set of SNPs, and LFDR.Small for LFDR from the small annotated category) as well as for which annotation category was used (EH_ext for Enhancer Hoffman extend 500, H3K9_Try for H3K9ac Trynka, H3K9_Try_ext for H3K9ac Trynka extend 500, EH for Enhancer Hoffman, H3K27_ext for H3K27ac PGC2 extend 500, H3K4_Try_ext for H3K4me3 Trynka extend 500, H3K27 for H3K27ac PGC2, H3K9 for H3K9ac peaks Trynka and FDHS for Fetal DHS Trynka). Differences between overall LFDR and maximum entropy LFDR are also provided (Diff). The gene name is provided if the SNP is in a gene.
(XLSX)
