# Supplementary figures and images for: The performance of a new local false discovery rate method on tests of association between coronary artery disease (CAD) and genome-wide genetic variants
Source: PLoS One. 2017 Sep 20;12(9):e0185174. doi: 10.1371/journal.pone.0185174 (PMC5607215; doi:10.1371/journal.pone.0185174)

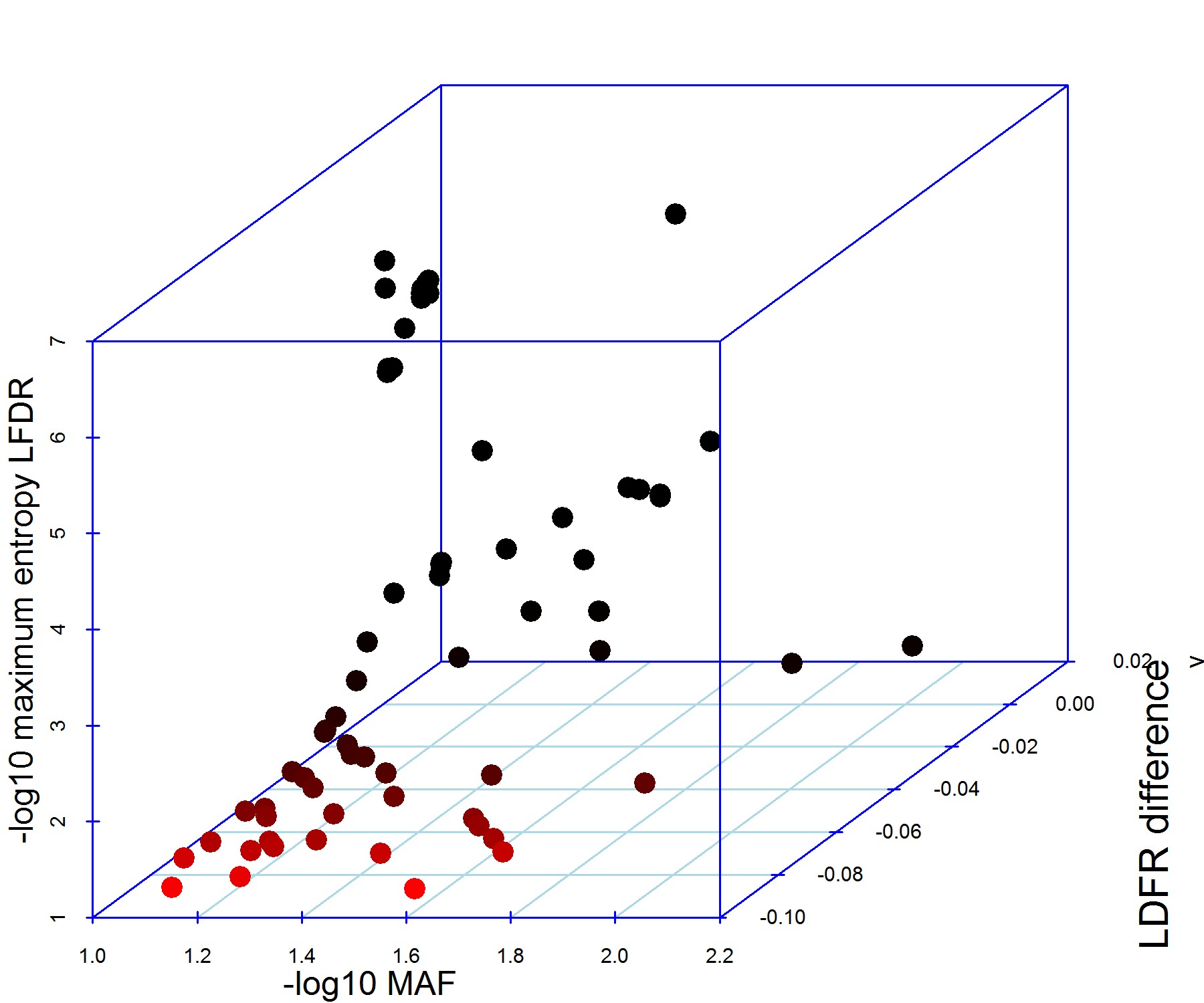

Supplement: S1 Fig — (TIF) [file pone.0185174.s001.tif]

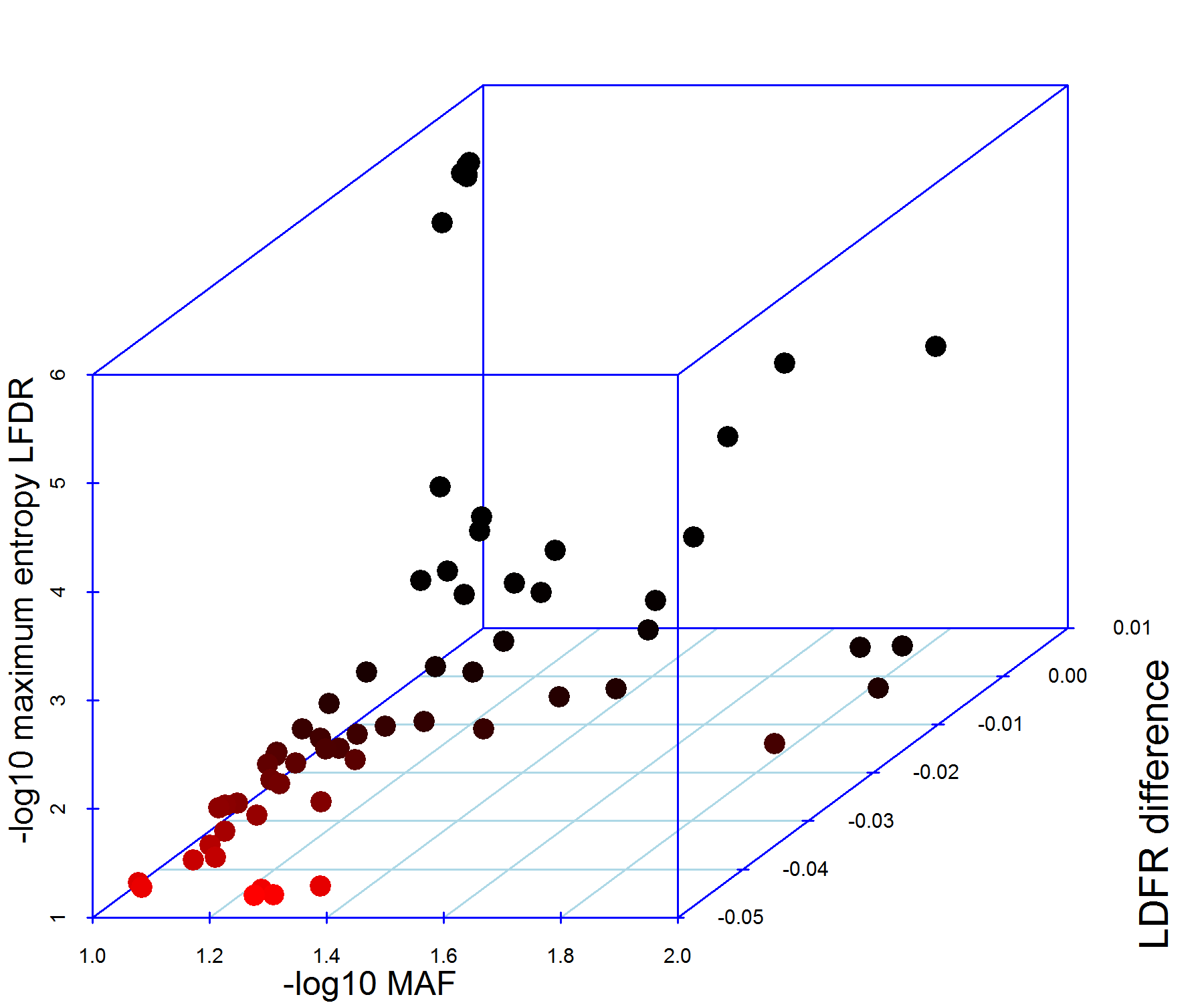

Supplement: S2 Fig — (TIF) [file pone.0185174.s002.tif]
